# New Advances in the Regulation of Leaf Senescence by Classical and Peptide Hormones

**DOI:** 10.3389/fpls.2022.923136

**Published:** 2022-06-28

**Authors:** Peixin Huang, Zhonghai Li, Hongwei Guo

**Affiliations:** ^1^Beijing Advanced Innovation Center for Tree Breeding by Molecular Design, National Engineering Research Center for Tree Breeding and Ecological Restoration, College of Biological Sciences and Technology, Beijing Forestry University, Beijing, China; ^2^Key Laboratory of Molecular Design for Plant Cell Factory of Guangdong Higher Education Institutes, Department of Biology, Southern University of Science and Technology, Shenzhen, China

**Keywords:** leaf senescence, plant hormones, peptide, senescence-associated gene, regulatory network

## Abstract

Leaf senescence is the last stage of leaf development, manifested by leaf yellowing due to the loss of chlorophyll, along with the degradation of macromolecules and facilitates nutrient translocation from the sink to the source tissues, which is essential for the plants' fitness. Leaf senescence is controlled by a sophisticated genetic network that has been revealed through the study of the molecular mechanisms of hundreds of senescence-associated genes (SAGs), which are involved in multiple layers of regulation. Leaf senescence is primarily regulated by plant age, but also influenced by a variety of factors, including phytohormones and environmental stimuli. Phytohormones, as important signaling molecules in plant, contribute to the onset and progression of leaf senescence. Recently, peptide hormones have been reported to be involved in the regulation of leaf senescence, enriching the significance of signaling molecules in controlling leaf senescence. This review summarizes recent advances in the regulation of leaf senescence by classical and peptide hormones, aiming to better understand the coordinated network of different pathways during leaf senescence.

## Introduction

Leaf senescence occurs as the final step of leaf development, preceding the ultimate cell death or completion of life cycle (Pennell and Lamb, 1997; Lim et al., 2007). Leaves are the important organ that can store energy in form of carbohydrate molecules converted from light energy through photosynthesis. As leaves age, photosynthetic efficiency declines and chloroplasts degrade, accompanied by the degradation of macromolecules such as lipids, proteins, and nucleic acids (Gan and Amasino, 1997; Lim et al., 2007). Leaf senescence is crucial for plant development and fitness because the nutrients released from senescent leaves are reallocated to other developing young organs for better reproductive growth in annual plants, or to be stored in phloem tissues for successful growth of next season in perennial plants (Cooke and Weih, 2005; Lim et al., 2007). Premature or delayed leaf senescence evidently reduces the yield and quality of crop plants such as rice and wheat (Buchanan-Wollaston et al., 2005; Srivalli and Khanna-Chopra, 2009; Breeze et al., 2011; Su et al., 2017; Piao et al., 2019). Studying regulatory mechanisms of leaf senescence will provide instructive hints for precise improvement of agronomic yield and quality.

The onset of leaf senescence depends mainly on the developmental age, as demonstrated by the fact that only plants older than 24 days induced significant yellowing on cotyledons after ethylene treatment, while plants younger than 17 days did not (Jing et al., 2002). It is also influenced by various endogenous and environmental factors. Out of them, plant hormones play pivotal roles in the regulation of leaf senescence. Basically, cytokinins (CKs), auxin, and gibberellins (GAs) delay leaf senescence, whereas ethylene, salicylic acid (SA), jasmonic acid (JA), abscisic acid (ABA), brassinosteroids (BRs), and strigolactones (SLs) accelerate senescence (Gan and Amasino, 1995; Li et al., 1996, 2013; Morris et al., 2000; Kim et al., 2011; Jibran et al., 2013; Yamada and Umehara, 2015; Zhu et al., 2015; Mao et al., 2017; Guo et al., 2021). Hormones not only directly regulate leaf senescence process, but also link environmental signals into the modulation of leaf senescence (Yang et al., 2011; Zhang et al., 2019). Briefly, plant hormones regulate leaf senescence through the following pathways: (i) affect leaf growth and development and alter the state of leaves that can be used to induce senescence; (ii) influence the progression and features of senescence via genetic transduction; (iii) integrate the environmental signals into developmental processes. Recently, peptide hormones were found to participate in the regulation of leaf senescence (Aghdam et al., 2021a; Zhang et al., 2022b), and studies on mechanism of senescence regulated by peptide hormones enrich the knowledge to understand regulation of leaf senescence. In this review, we provide an overview and highlight new advances in the regulation of leaf senescence by classical and peptide hormones. Major components of hormones biosynthesis and signaling involving in leaf senescence are presented (Table 1).

**Table 1:** List of the key components in hormone pathway involved in leaf senescence.

**Gene**	**Hormone**	**Effect**	**Species**	**Reference**
*IPT*	CK	Delay	*Arabidopsis thaliana*	Gan and Amasino, 1995
*FPS1S*	CK	Promote	*Arabidopsis thaliana*	Masferrer et al., 2002
*SlymiRNA208*	CK	Promote	*Solanum lycopersicon*	Zhang et al., 2020b
*APT1*	CK	Promote	*Arabidopsis thaliana*	Zhang et al., 2013b
*AHK3*	CK	Delay	*Arabidopsis thaliana*	Kim et al., 2006
*AHK2, AHK4*	CK	Delay	*Arabidopsis thaliana*	Riefler et al., 2006
*ARR2*	CK	Delay	*Arabidopsis thaliana*	Kim et al., 2006
*CRF6*	CK	Delay	*Arabidopsis thaliana*	Zwack et al., 2013
*YUC6*	Auxin	Delay	*Arabidopsis thaliana*	Kim et al., 2011
*ZmGH3.8*	Auxin	Delay	*Zea mays*	Feng et al., 2021
*IAA17*	Auxin	Promote	*Arabidopsis thaliana*	Shi et al., 2015
*ARF2*	Auxin	Promote	*Arabidopsis thaliana*	Lim et al., 2010
*ARF1, ARF7, ARF19*	Auxin	Promote	*Arabidopsis thaliana*	Ellis et al., 2005
*ANT*	Auxin	Delay	*Arabidopsis thaliana*	Feng et al., 2016
*SAUR36*	Auxin	Promote	*Arabidopsis thaliana*	Hou et al., 2013
*SAUR39*	Auxin	Promote	*Oryza sativa*	Kant et al., 2009
*SAUR49*	Auxin	Promote	*Arabidopsis thaliana*	Wen et al., 2020
*BrGA20ox3*	GA	Delay	*Brassica rapa*	Xiao et al., 2019
*GAI, RGA, RGL1, RGL2*	GA	Delay	*Arabidopsis thaliana*	Chen et al., 2014
*ScGAI*	GA	Delay	*Saccharum spp*.	Fang et al., 2021
*ACS*	Ethylene	Promote	*Arabidopsis thaliana*	Tsuchisaka et al., 2009
*ETR1, ERS1*	Ethylene	Delay	*Arabidopsis thaliana*	Qu et al., 2007
*EIN2*	Ethylene	Promote	*Arabidopsis thaliana*	Oh et al., 1997
*EIN3, EIL1*	Ethylene	Promote	*Arabidopsis thaliana*	Chao et al., 1997
*miRNA164*	Ethylene	Delay	*Arabidopsis thaliana*	Li et al., 2013
*ORE1*	Ethylene	Promote	*Arabidopsis thaliana*	Kim et al., 2009
*ORS1, AtNAP, ANAC019/047/055*	Ethylene	Promote	*Arabidopsis thaliana*	Kim et al., 2014
*ZmNAC126*	Ethylene	Promote	*Zea mays*	Yang et al., 2020
*ERF4, ERF8*	Ethylene	Promote	*Arabidopsis thaliana*	Koyama et al., 2013
*SID2*	SA	Promote	*Arabidopsis thaliana*	Abreu and Munne-Bosch, 2009
*PAD4*	SA	Promote	*Arabidopsis thaliana*	Morris et al., 2000
*WHY1*	SA	Delay	*Arabidopsis thaliana*	Lin et al., 2020
*S3H*	SA	Delay	*Arabidopsis thaliana*	Zhang et al., 2013a
*S5H/DMR6*	SA	Delay	*Arabidopsis thaliana*	Zhang et al., 2017b
*NPR1*	SA	Promote	*Arabidopsis thaliana*	Morris et al., 2000
*PVA31*	SA	Promote	*Arabidopsis thaliana*	Ichikawa et al., 2015
*LOX1, LOX2, LOX3, LOX4*	JA	Promote	*Arabidopsis thaliana*	He et al., 2002
*KAT2*	JA	Promote	*Arabidopsis thaliana*	Castillo and Leon, 2008
*TaWRKY13-A*	JA	Promote	*Triticum aestivum*	Qiao et al., 2021
*TaWRKY42-B*	JA	Promote	*Triticum aestivum*	Zhao et al., 2020
*miR139*	JA	Delay	*Arabidopsis thaliana*	Schommer et al., 2008
*TCP2, TCP4, TCP10*	JA	Promote	*Arabidopsis thaliana*	Schommer et al., 2008
*COI1*	JA	Promote	*Arabidopsis thaliana*	Castillo and Leon, 2008
*COS1*	JA	Delay	*Arabidopsis thaliana*	Xiao et al., 2004
*JAZ7*	JA	Delay	*Arabidopsis thaliana*	Yu et al., 2016
*MYC2, MYC3, MYC4*	JA	Promote	*Arabidopsis thaliana*	Zhu et al., 2015; Yu et al., 2016
*Dof2.1*	JA	Promote	*Arabidopsis thaliana*	Zhuo et al., 2020
*OsERF101*	JA	Promote	*Oryza sativa*	Lim et al., 2020
*MdBT2, MdJAZ2*	JA	Delay	*Malus pumila Mill*.	An et al., 2021a
*ESR/ESP*	JA	Delay	*Arabidopsis thaliana*	Miao and Zentgraf, 2007
*PSF*	ABA	Delay	*Oryza sativa*	Wang et al., 2016
*ES3(t)*	ABA	Delay	*Oryza sativa*	Su et al., 2017
*PvCCCH69*	ABA	Delay	*Panicum virgatum*	Xie et al., 2021
*CsHB5*	ABA	Promote	*Citrus reticulata Blanco*.	Zhang et al., 2021d
*OsNAC2*	ABA	Promote	*Oryza sativa*	Mao et al., 2017
*CDF4*	ABA	Promote	*Arabidopsis thaliana*	Xu et al., 2020
*OsMYB102*	ABA	Delay	*Oryza sativa*	Piao et al., 2019
*AAO3*	ABA	Promote	*Arabidopsis thaliana*	Yang et al., 2014
*SAG113*	ABA	Promote	*Arabidopsis thaliana*	Zhang et al., 2012
*ABA2*	ABA	Delay	*Arabidopsis thaliana*	Song et al., 2016
*PYL8*	ABA	Promote	*Arabidopsis thaliana*	Lee et al., 2015
*PYL9*	ABA	Promote	*Arabidopsis thaliana*	Zhao et al., 2016
*ABIG1*	ABA	Promote	*Arabidopsis thaliana*	Liu et al., 2016
*ABF2, ABF3, ABF4*	ABA	Promote	*Arabidopsis thaliana*	Gao et al., 2016
*OsNAP*	ABA	Promote	*Oryza sativa*	Liang et al., 2014
*ONAC054*	ABA	Promote	*Oryza sativa*	Sakuraba et al., 2020
*ABI5*	ABA	Promote	*Arabidopsis thaliana*	Su et al., 2016
*DET2*	BR	Promote	*Arabidopsis thaliana*	Li et al., 1996
*UGT73C6*	BR	Delay	*Arabidopsis thaliana*	Husar et al., 2011
*CYP105A1*	BR	Delay	*Arabidopsis thaliana*	Dasgupta et al., 2011
*DRL1/BAT1*	BR	Delay	*Agrostis stolonifera L*.	Han et al., 2017
*BRI1*	BR	Promote	*Arabidopsis thaliana*	Li and Chory, 1997
*BES1*	BR	Promote	*Arabidopsis thaliana*	Yin et al., 2002
*AIF2*	BR	Delay	*Arabidopsis thaliana*	Kim et al., 2020
*CCD7*	SL	Promote	*Lotus japonicus*	Liu et al., 2013
*CCD8*	SL	Promote	*Petunia hybrida*	Snowden et al., 2005
*ORE9/MAX2*	SL	Promote	*Arabidopsis thaliana*	Woo et al., 2001
*CLE14*	Peptide	Delay	*Arabidopsis thaliana*	Zhang et al., 2022b
*CLE42*	Peptide	Delay	*Arabidopsis thaliana*	Zhang et al., 2022a
*PSKR1*	Peptide	Delay	*Arabidopsis thaliana*	Matsubayashi et al., 2006
*TPST*	Peptide	Delay	*Arabidopsis thaliana*	Komori et al., 2009

## Plant Hormones That Delay Leaf Senescence

### Cytokinins

Cytokinins (CKs) are N^6^-substituted adenine derivatives that regulate diverse aspects of plant growth and development processes, including shoot meristems, vascular development, root growth, nodulation, as well as leaf initiation and leaf senescence (Argueso et al., 2010; Perilli et al., 2010; Wu et al., 2021). CK works as a negative regulator of senescence, which was supported by the evidences that exogenous application CK retards senescence and endogenous CKs decrease during senescence (Singh et al., 1992; Gan and Amasino, 1996). Expressions of CK synthesis-associated genes decrease and a CK oxidase involving CK degradation is up-regulated when the senescence occurs, which is consistent with the gradually decrease of CKs content along with senescence (Buchanan-Wollaston et al., 2005). The expression of isopentenyl transferase (*IPT*) driven by a senescence-specific promoter *SAG12* obviously delays leaf senescence process (Gan and Amasino, 1995). The autoregulatory *proSAG12:IPT* system has been widely utilized in numerous important crop plants that exhibit retarded leaf senescence, indicative of the negative regulation of leaf senescence by CK. Therefore, the researchers manipulated the leaf senescence process by regulating the CK content through molecular genetic pathways. Overexpression of *FPS1S* (Farnesyl diphosphate synthase) leads to the declined endogenous zeatin-type CK with a concomitant senescence-like phenotype in *Arabidopsis* (Masferrer et al., 2002). SlymiRNA208 suppresses the post-transcriptional expression of *SlIPT2* and *SlIPT4* in tomato, resulting in the premature leaf senescence in the *SlymiRNA208*-overexpressing plants by reducing the endogenous concentration of CKs (Zhang et al., 2020b). APT1 (Adenine phosphoribosyl transferase 1), which catalyzes the CK conversion from free bases to nucleotides, acts as a positive regulator of leaf senescence. Loss of APT1 activity causes a delayed leaf senescence due to the excess accumulation of CKs (Zhang et al., 2013b).

The CK signaling pathway initiates with the binding of CKs to histidine kinase receptors, known as AHK2, AHK3, and CRE1/WOL/AHK4, then involves a phosphotransfer cascade, and ultimately triggers transcription of CK-responsive genes in the nucleus (Argueso et al., 2010). Several components of CK signal transduction are found to function in leaf senescence (Table 1). Gain-of-function of AHK3 leads to the extended leaf longevity, conversely *ahk3* loss-of-function mutant exhibits early leaf senescence during dark-induced senescence (Kim et al., 2006). AHK3 mediates the specific phosphorylation of downstream type-B response regulator ARR2 that plays a crucial role in CK-mediated leaf longevity. Consistently, the plants overexpressing *ARR2* show delayed leaf senescence during dark-induced and age-dependent senescence, but not overexpression of ARR2^D80N^, in which the phosphotransfer to ARR2 is abolished (Hwang and Sheen, 2001; Kim et al., 2006). Interestingly, the inhibition of ARR2 degradation through a substitution of Lys90 with Gly also delays leaf senescence (Kim et al., 2012). Although AHK3 plays a major role in CK-dependent chlorophyll retention in the detached leaves, AHK2 and AHK4 also contribute to CKs-mediated leaf longevity (Riefler et al., 2006). Cytokinin response factors (CRFs) are transcriptionally induced by CK and act downstream of AHK3 to regulate leaf senescence. Plants with overexpressing *CRF6* possess a higher chlorophyll retention than wild type without exogenous CK (Zwack et al., 2013), unraveling CRF6 as a negative regulator during dark-induced leaf senescence. In contrast, simultaneous silencing of CRF1/3/5/6 delays leaf senescence and overexpression of CRF1/3/5 accelerates senescence, accompanied by an induction of *SAG12* and reduction of *CAB2* (Raines et al., 2016). The different roles in regulating leaf senescence imply that CRFs are involved in regulating the leaf senescence process through different pathways downstream of CK.

CKs participate in the regulation of sink/source relations during leaf senescence, which partially depends upon the activity of cell-wall invertase (CWINV) (Godt and Roitsch, 1997; Balibrea Lara et al., 2004). CWINV and hexose transporters are effective enzymes in mediating the phloem unloading process of sucrose. Expression of CWINV under the control of *SAG12* promoter (*proSAG12:CWINV*) delays leaf senescence (Godt and Roitsch, 1997; Balibrea Lara et al., 2004). Further, a link between CK and CWINV underlying leaf senescence is substantiated by the evidence that expression of an invertase inhibitor driven by a CK-inducible promoter does not show delayed senescence in the presence of CKs (Balibrea Lara et al., 2004; Jin et al., 2009). Taken together, CWINV is an essential component that mediates CK-conferred leaf longevity. Besides, CKs influence leaf senescence via interaction with other hormones. For example, CKs reduce ABA content through suppressing the transcription of ABA synthesis-related genes but elevating the expression of ABA degradation genes, which leads to the retarded leaf senescence (Zhang et al., 2021c). Given the regulatory mechanism of CKs in sink/source relations is largely unknown, more investigations needs to be done to unravel the role of CKs in the regulation of leaf senescence, especially regarding sink/source relations, which will be useful for application in molecular breeding.

### Auxin

Auxin not only functions in cell growth in response to environmental stimuli, but also fulfills an important role in leaf initiation, morphogenesis, as well as senescence (Vanneste and Friml, 2009). Indole-3-acetic acid (IAA), a major natural auxin, transiently inhibits the expression level of *SAG12* (Noh and Amasino, 1999), indicating that exogenous auxin represses leaf senescence process. Surprisingly, the endogenous IAA levels detected in senescent leaves were 2-fold higher than in fully expanded young leaves (Quirino et al., 1999; van der Graaff et al., 2006). Although this increase may be due to non-uniform regulation between cells in the senescent and non-senescent parts, the significance of this increase during leaf senescence is not clear. A recent study on the effect of IAA on gene expression during leaf senescence reported that IAA treatment accelerated the progression of senescence-related changes, and furthermore, it revealed that the earlier treatment time, i.e. 27 DAS (a few days after sowing), resulted in the most significant acceleration of late leaf senescence compared to 35 DAS (Goren-Saglam et al., 2020). Further microarray analysis of gene expression of IAA treatment at different time points showed that the effect of IAA on leaf senescence was not only time-dependent but also interacted with ethylene and JA pathways. Therefore, discussion of the function of IAA in leaf senescence requires consideration of the balance of endogenous hormone networks.

Activating or mutating components of the auxin pathway in planta helps us to better understand the function of IAA in leaf senescence. YUCCAs (YUCs) encoding flavin monooxygenases catalyze a restrictive step in auxin biosynthesis, namely the conversion from indole-3-pyruvic acid (IPA) to auxin (Kim et al., 2007). Activation of YUC6 in *Arabidopsis* increases free IAA levels, reduces expression of SAGs and exhibits a delayed senescence phenotype (Kim et al., 2011). The thiol-reductase activity of YUC6 also mediates reactive oxygen species (ROS) content and auxin availability to influence leaf senescence (Cha et al., 2016). Auxin signal is perceived by its receptor protein TRANSPORT INHIBITOR RESPONSE 1 (TIR1)/AUXIN SIGNALING F BOX PROTEINs (AFBs), leading to the ubiquitin-mediated degradation of AUX/IAA proteins, which are repressors of auxin response factors (ARFs) (Vanneste and Friml, 2009). Then activation of auxin response genes is accompanied by the release of ARFs. The plant overexpressing signaling component AtIAA17 displays early leaf senescence with lower chlorophyll content in rosette leaves, conversely *iaa17* mutant shows a delayed senescence phenotype (Shi et al., 2015). ARF2, a transcriptional repressor of auxin signaling, is induced in senescing leaves. The *arf2* mutant displays the delayed senescence symptoms of rosette leaves in natural and dark conditions (Lim et al., 2010). AINTEGUMENTA (ANT), a member of the AP2/ERF TF family, is demonstrated to act downstream of ARF2 to extend leaf longevity (Feng et al., 2016). Moreover, mutations in *ARF7* and *ARF19*, two transcriptional activators, enhance *arf2* phenotype (Ellis et al., 2005), which indicates that auxin is involved in the regulation of leaf senescence by controlling gene expression in manifold ways. The early auxin-responsive genes, including *SMALL AUXIN-UP RNA* (*SAURs*) genes such as *SAUR36, SAUR39*, and *SAUR49*, are involved in leaf senescence (Kant et al., 2009; Hou et al., 2013; Wen et al., 2020). The soybean (*Glycine max*) SENESCENCE-ASSOCIATED RECEPTOR-LIKE KINASE (GmSARK) and its ortholog in *Arabidopsis* AtSARK act as positive regulators of leaf senescence through a widespread mechanism relating to auxin, ethylene, and cytokinin (Xu et al., 2011). SAUR49 promotes leaf senescence via activation of SARK-mediated signaling by repressing SENESCENCE SUPPRESSED PROTEIN PHOSPHATASE (SSPP) (Wen et al., 2020). Therefore, SAURs-SARK regulation mode may be significant for integrating the senescence signals and hormone signaling in plants. Recently, modification of autophagy and auxin signals via manipulating expression of *ZmATG18b* and *ZmGH3.8* gene alters the time of maize leaf senescence (Feng et al., 2021). Besides, IAA29 is involved in the PIF4 and PIF5-mediated regulation of heat stress-induced leaf senescence (Li et al., 2021b). Taken together, these studies suggest that auxin may interact with other hormones and environmental signals to coordinate plant growth and the onset of leaf senescence at the right time.

### Gibberellins

Gibberellins (GAs) are a class of tetracyclic diterpenoid, some of which are bioactive in regulating many aspects of plant growth and development, such as stem elongation, leaf expansion, seed dormancy and germination, plant flowering, and response to abiotic and biotic stresses (Gao and Chu, 2020). The content of endogenous GAs declines as leaves age, and exogenous application of GA_3_ retards the degradation of chlorophyll (Aharoni, 1978; Li et al., 2010), indicating that GAs repress the progression of senescence. The GA 2-oxidase 2 (GA2OX2) gene, which causes GA inactivation, is up-regulated 18-fold during leaf senescence (van der Graaff et al., 2006), suggesting that the decrease in active GA may be a cause of leaf senescence. TEOSINTE BRANCHED1/CYCLOIDEA/PCF (TCP) TF BrTCP21 directly binds the promoter of GA biosynthetic gene *BrGA20ox3*, activates its transcription, and delays leaf senescence (Xiao et al., 2019). The transcript of *BrTCP21* decreases along with leaf senescence, while GA_3_ treatment keeps *BrTCP21* expression in a higher level, which suggests that the positive feedback loop of GA-BrTCP21-GA plays an important role in leaf senescence. In *Arabidopsis*, GA signal is received by the receptor GID1 and then transduced to release the repression of TFs by negative regulator DELLA proteins, including GAINSENSITIVE (GAI), REPRESSOR OF GA1-3 (RGA), RGA-LIKE1 (RGL1), RGA-LIKE2 (RGL2), and RGA-LIKE3 (RGL3) (Olszewski et al., 2002; Hedden and Sponsel, 2015). The natural leaf senescence occurs earlier when four DELLA proteins (*gai-t6 rga-t2 rgl1-1 rgl2-1*) are knocked-out (Chen et al., 2014), which suggests what appears to be a contradiction with GA inhibition of leaf senescence. Since GA is an important regulator of plant flowering and the *gai-t6 rga-t2 rgl1-1 rgl2-1* mutant has an early flowering phenotype, it cannot be simply assumed that GA promotes senescence. Therefore, the functions of DELLA and GA cannot be equated in the regulation of leaf senescence. In supporting this hypothesis, DELLA proteins delay leaf senescence by interacting with and suppressing the functions of WRKY45, WRKY6, WRKY75, and NAP, positive regulators of leaf senescence (Chen et al., 2017; Zhang et al., 2018a, 2021b; Lei et al., 2020). Similarly, ScGAI delays age-trigged senescence by interacting with and then repressing the function of ScNAC23 in sugarcane (Fang et al., 2021). Additionally, GAs might indirectly regulate leaf senescence via crosstalk with other hormones. The decline of GA level is usually accompanied by an increase of ABA content, and exogenous GA_3_ treatment could inhibit the surge of ABA during leaf senescence (Yu et al., 2009), implying an antagonistic effect of GA and ABA in regulation of leaf senescence.

## Plant Hormones That Accelerate Leaf Senescence

### Ethylene

Ethylene is a well-known gas phytohormone that acts as an endogenous facilitator of plant aging, including the senescence processes of leaf and petal, as well as fruit ripening. Exogenous application of ethylene or increase in endogenous ethylene content promotes leaf senescence, while inhibitors of ethylene biosynthesis retard senescence (Abeles et al., 1988; Grbić and Bleecker, 1995; Wang et al., 2001). Ethylene does not directly determine the onset of leaf senescence, since ethylene only accelerates the progression of leaf senescence when leaves reach a defined age (Jing et al., 2002, 2005). Transcription analysis reveals that a number of genes involving ethylene biosynthesis and signaling components are regulated in senescent leaves (van der Graaff et al., 2006). 1-aminocyclopropane-1-carboxylate (ACC) synthases (ACS) are biosynthetic enzymes to produce the key precursor of ethylene, and the *acs octuple* mutant exhibits a prominently delayed senescence (Tsuchisaka et al., 2009). The role of ethylene in the control of leaf senescence is also explained by the function of signaling elements, as evidenced by the *etr1-1, ein2*, and *ein3 eil1* mutants with extended leaf longevity (Grbić and Bleecker, 1995; Chao et al., 1997; Oh et al., 1997), and *etr1 ers1* with earlier senescence (Qu et al., 2007), which is consistent with the positive effect of ethylene in regulating leaf senescence.

ETHYLENE-INSENSITIVE2 (EIN2) is a central positive regulator of ethylene signaling and mediates most of the ethylene response. Expression of *ORE1/NAC092*, one target of miR164, is up-regulated by EIN2 during gradually leaf aging, while *miR164* expression is suppressed by EIN2 (Kim et al., 2009). The trifurcate feed-forward pathway involving ORE1, miR164 and EIN2 finally results in increased expression of *ORE1* that promotes leaf senescence. ETHYLENE-INSENSITIVE3 (EIN3) is a master transcription factor in ethylene signaling, and acts downstream of EIN2 to regulate ethylene response. EIN3 represses *miR164* transcription via directly binding the promoter of *miR164*, leading to increased transcript levels of *ORE1* (Li et al., 2013). The linear pathway involving EIN2-EIN3-miR164-ORE1 sheds light on accelerated leaf senescence by ethylene regulation. However, EIN2 does not fully depend on ORE1 in regulating senescence, since EIN2 still contributes to senescence-associated cell death in the absence of ORE1. More senescence-associated NAC transcription factors are found to act as the downstream components of EIN2 governing leaf senescence, including ANAC019, AtNAP, ANAC047, ANAC055, and ORS1 (Kim et al., 2014). WRKY71 functions as a positive regulator of leaf senescence by communicating with ethylene signal in *Arabidopsis*. WRKY71 is an ethylene-inducible gene and influences leaf senescence by directly regulating EIN2 and ORE1 (Yu et al., 2021b). EIN3 and ORE1 induce the chlorophyll degradation through directly activating chlorophyll catabolic genes (Qiu et al., 2015). ZmNAC126 is transactivated by ZmEIN3 and regulates chlorophyll degradation of ethylene-induced senescence in maize (Yang et al., 2020). Therefore, an intricate transcription network involving EIN2 and EIN3 plays significant roles in ethylene-triggered leaf senescence. AP2/ERF transcription factors such as AtERF4 and AtERF8, activated by ethylene, also involve in modulating the onset of leaf senescence (Koyama et al., 2013; Koyama, 2014). Additionally, ethylene interacts with other hormones to influence leaf senescence (Kim et al., 2015; Iqbal et al., 2017). For example, the detached leaves of *ein2* and *ein3 eil1* mutants are insensitive to MeJA-induced leaf senescence compared to that in wild type (Li et al., 2013), indicating that JA-induced leaf senescence is dependent upon ethylene signal. Ethylene is thought to be a downstream signal that promotes the progression of leaf senescence in an age-dependent manner. However, the mechanisms involved are not fully understood. Figuring out the relationship between ethylene and age information and how age information is encoded will really help to understand the nature of leaf senescence.

### Salicylic Acid

Salicylic acid (SA) is a phenolic hormone involved in plant development, abiotic and biotic stress adaption. It is critical for defense against plant pathogens, especially as a component of systemic acquired resistance (Malamy et al., 1990; Metraux et al., 1990). SA content gradually increases in the senescing leaves of multiple species (Zhang et al., 2017b), and SA deficiency mediated by transgenic *NahG* line and defect in *SID2*, an isochorismate synthase of SA biosynthesis, delays leaf senescence in *Arabidopsis* (Abreu and Munne-Bosch, 2009), depicting a connection between SA and leaf senescence. The intact SA signaling pathway contributes to control the expression of senescence-enhancing genes whose increased transcripts are disrupted by mutations in NPR1 or PAD4 (Morris et al., 2000). PHYTOALEXIN DEFICIENT4 (PAD4) promotes SA accumulation, especially in response to pathogen infection (Makandar et al., 2015). PAD4-dependent SA pathway has a central role in *saul1* mutant-mediated initiation of leaf senescence to induce visible symptoms of senescence, and activation of senescence in the aphid-infested leaves (Pegadaraju et al., 2005; Vogelmann et al., 2012). Leaf senescence induced by SA is associated with SA-dependent cell death, since *pad4* mutant exhibits a delayed yellowing and reduced necrosis at the final stage of senescence (Morris et al., 2000). The retrograde signaling protein WHIRLY1 (WHY1) alters its organelle isoforms in nucleus or chloroplasts, and perturbs SA homeostasis via regulating expression of SA biosynthesis genes *SID2* and *PAL1* (Lin et al., 2020). SA 3-hydroxylase (S3H) and S5H are involved in the SA catabolism by catalyzing SA conversion into 2,3- and 2,5-dihydroxybenzoic acid, and the defect in S3H and S5H over-accumulates active SA content, leading to an early senescence (Zhang et al., 2013a, 2017b). Thus, the active SA content, accompanied by regulation of SA homeostasis plays an essential role in promoting leaf senescence. A number of WRKY TFs are unraveled to influence leaf senescence via modulating SA pathway by different modes. WRKY75, WRKY28, WRKY55, WRKY40, WRKY46, WRKY51, WRKY60, and WRKY63 activate the expression of *SID2* by binding to its promoter, thus augment the accumulation of SA to accelerate leaf senescence (Guo et al., 2017; Zhang et al., 2017a; Tian et al., 2020; Wang et al., 2020). WRKY TFs are also able to affect senescence through indirectly regulating the biosynthesis and signaling of SA, for example, WRKY28 mediates SA biosynthesis in response to light signals (Tian et al., 2020). WRKY46 also interacts with NPR1, the core component of SA signal transduction, to improve *WRKY6* expression to mediate probenazole/SA-elicited leaf senescence (Zhang et al., 2021a).

SA pathway integrates many signals or physiological processes to change states of leaf senescence. Recently, two groups simultaneously reveal that SA coordinates with ethylene to accelerate leaf senescence, achieved by interaction of NPR1 and EIN3 to promote expression of SAGs synergistically (Wang et al., 2021; Yu et al., 2021a). In addition, SA signaling is involved in leaf senescence induced by autophagy, PVA31-mediated membrane trafficking, membrane phospholipid metabolism, and ROS (Yoshimoto et al., 2009; Xiao et al., 2010; Ichikawa et al., 2015; Guo et al., 2017). In summary, SA plays an important role in the onset and development of leaf senescence and is coordinated by multiple factors. Since SA mediates both immunity and aging, SA is the best link to explore the relationships between immunity and senescence.

### Jasmonic Acid

Jasmonic acid (JA) is a class of oxylipin phytohormones derived from polyunsaturated fatty acids, preferentially α-linolenic acid (Li et al., 2021a). JA regulates myriad aspects of plant growth and development, as well as stress responses. JA accumulates in senescing leaves and positively regulates leaf senescence (He et al., 2002). JA biosynthesis-associated genes are differentially regulated during leaf senescence, including *LOX, AOS, AOC*, and thiolase (He et al., 2002). *LOX1, LOX3*, and *LOX4* are obviously up-regulated with the progression of leaf senescence, while *LOX2* is down-regulated (He et al., 2002). This difference implies different roles for LOXs in regulating senescence, such as the exclusive role of LOX2 in stress-induced leaf senescence (Seltmann et al., 2010). *KAT2* (*3-ketoacyl-CoA thiolase 2*), a gene encoding the JA-biosynthetic β-oxidation enzyme, is strongly activated in natural and dark-induced senescing leaves, while reduction of *KAT2* expression leads to significantly delayed senescence (Castillo and Leon, 2008). Several factors involve in regulation of leaf senescence through modulating JA biosynthesis. TaWRKY13-A and TaWRKY42-B facilitate the onset and progression of leaf senescence by promoting the expression of *LOX* genes, which consequently induces accumulation of JA content in wheat (Zhao et al., 2020; Qiao et al., 2021). miR139 indirectly controls JA biosynthesis via changing TCPs activity, thus overexpression of *miR139* delays leaf senescence (Schommer et al., 2008).

Components of JA signaling pathway take part in the regulation of leaf senescence. JA perception is achieved by receptors complex comprised of COI1 (CORONATINE INSENSITIVE1) and JAZ family proteins, subsequentially leading to the degradation of JAZ proteins via 26S-proteosome (Li et al., 2021a). JA-induced premature senescence is blocked in JA insensitive mutant *coi1* (He et al., 2002; Castillo and Leon, 2008), suggesting the intact JA pathway is necessary for senescence activation. Xiao et al. (2004) screened for the suppressors of *coi1* and isolated the *cos1* (*coi1 suppressor1*) mutant. Defect in *COS1* gene, which encodes lumazine synthase for riboflavin pathway, severely reduces the higher chlorophyll content in the *coi1* mutant compared with wild type (Xiao et al., 2004). The constant yellowing phenotype of *cos1 coi1* leaves points out a novel function of riboflavin pathway in regulating leaf senescence. As a negative regulator of JA signaling, JAZ7 is induced by darkness, thus disturbs the functions of downstream MYC2/MYC3/MYC4 TFs to suppress dark-induced leaf senescence (Yu et al., 2016). Meanwhile, the *jaz7* mutant exhibits more severe leaf yellowing and chlorophyll degradation (Yu et al., 2016). MYC2/MYC3/MYC4 are the master TFs of JA signaling pathway mediating JA-induced leaf senescence. JA-modulated leaf senescence is demonstrated to associate with regulation of ROS and chlorophyll degradation. For example, MYC2 represses the expression of *CATALASE 2* (*CAT2*) gene in JA-treated leaves, and the subsequent H_2_O_2_ accumulation leads to advanced leaf senescence (Zhang et al., 2020a). CAT2 mutation correctly rescuing delayed leaf senescence of *myc2* mutant further proves the important roles of ROS in JA-induced senescence. Moreover, MYC2/MYC3/MYC4 promote expression of chlorophyll catabolic genes, such as *PAO, NYC1*, and *NYE1*, by directly binding to their promoters, respectively, thus causing chlorophyll degradation, so that *myc2 myc3 myc4* exhibits a severe stay-green phenotype (Zhu et al., 2015). Additionally, MYCs proteins are not the only TFs participating in JA-regulated leaf senescence. ANAC019/055/072 exerts synergistic effects and bHLH subgroup IIId factors act antagonistically with MYCs to regulate JA-related leaf senescence (Qi et al., 2015; Zhu et al., 2015). Interestingly, the MYC2-Dof2.1-MYC2 feedforward transcriptional loop positively regulates dark-induced and age-dependent leaf senescence (Zhuo et al., 2020). In addition, the key components of JA signaling are also targeted by other factors to regulate leaf senescence process. For example, OsERF101 elevates the expression of *OsMYC2* and *OsCOI1a* to promote JA-mediated leaf senescence in rice (Lim et al., 2020). Apple MdBT2, a scaffold protein having ubiquitination activity, accelerates MdMYC2 degradation and stabilizes MdJAZ2 protein through direct interactions, thereby antagonistically regulates JA-activated leaf senescence (An et al., 2021a). Crosstalk between circadian clock and JA pathway finely cooperates many processes of plant growth and development. The Evening Complex (EC) in circadian oscillator negatively regulates JA-mediated leaf senescence via repressing the expression of *MYC2* (Zhang et al., 2018b, 2019). Furthermore, JA integrates with other endogenous phytohormones to affect leaf senescence. WRKY57 acts as a suppressor of JA-induced leaf senescence. Auxin antagonizes JA-induced leaf senescence process via up-regulating expression of WRKY57 (Jiang et al., 2014). Additionally, JAZ4/8 and IAA29, repressors of the JA and auxin signaling pathways respectively, competitively interact with WRKY57 (Jiang et al., 2014). Therefore, WRKY57 functions as an important integrator of JA and auxin pathways in leaf senescence modulation. JA-inducible ESR/ESP inhibits the functions of WRKY53, a positive regulator of leaf senescence. ESR/ESP and WRKY53 mediate leaf senescence on the basis of JA and SA homeostasis and the consequent regulation of these two genes antagonistically (Miao and Zentgraf, 2007). Autophagy up-regulated by low concentration SA alleviates JA-induced leaf senescence (Yin et al., 2020), further indicating a compact antagonistic interaction of SA and JA in leaf senescence. In summary, JA acts as an important integrative signal that communicates with other plant hormones to regulate leaf senescence and adjust the response to biotic and abiotic factors.

### Abscisic Acid

Abscisic acid (ABA) is a kind of phytohormone constituted of sesquiterpenoid. ABA regulates a myriad of plant development processes, such as seed dormancy and germination, stomatal closure, shoot and root growth, leaf senescence, as well as abiotic responses (Chen et al., 2020; Sano and Marion-Poll, 2021). ABA induces premature leaf senescence, and endogenous ABA content is an important regulatory factor affecting leaf senescence. Phenotype of rice *psf* (*premature senescence of flag leaves*) mutant that exhibits premature senescence lesion in senescent leaves results from high level of ABA accumulation, concomitantly with low rate of D1 protein synthesis and aggravated PSII photodamage during leaf senescence (Wang et al., 2016). The level of ABA content increases in the *early senescence 3* (*es3*) mutant, leading to the upregulation of SAGs (Su et al., 2017). Zinc finger protein PvCCCH69 suppresses age- and dark-induced leaf senescence via antagonizing ABA pathway (Xie et al., 2021). Several other factors modulate leaf senescence with regulation of ABA biosynthesis and metabolism. CsHB5, OsNAC2, and CDF4 elevate the expression of ABA biosynthetic genes, increase ABA content, and promote leaf senescence (Mao et al., 2017; Xu et al., 2020; Zhang et al., 2021d). OsMYB102 and OsNAC2 are involved in ABA metabolism via regulating expression of ABA catabolic enzymes (Mao et al., 2017; Piao et al., 2019). OsMYB102 inhibits ABA accumulation by inducing ABA catabolic gene *OsCYP707A6*, thereby delaying age- or dark-induced leaf senescence (Piao et al., 2019). NAP promotes chlorophyll degradation by upregulating ABA biosynthetic gene *AAO3*, and accelerates leaf senescence (Yang et al., 2014). Moreover, the ABA-NAP-SAG113 module controls stomatal movement and water loss during leaf senescence (Zhang and Gan, 2012; Zhang et al., 2012). In view of ABA actions in leaf senescence, it is thought that loss function of ABA biosynthetic genes should cause a delayed senescence. On the contrary, mutation in ABA2 (*aba2/eas1*) decreases ABA content but accelerates leaf senescence (Pourtau et al., 2004; Song et al., 2016). This opposite role may be explained by the role of ABA in both cytoprotective and senescence activities, and the function of ABA in influencing leaf yellowing is accurately balanced by both processes depending on plant age or environmental conditions.

In addition to the endogenous ABA content, ABA signaling pathway also plays essential roles in regulation of leaf senescence. As members of the receptors for ABA signaling, plants overexpressing P*YL8* and *PYL9* exhibit enhanced dark-induced or ABA-induced leaf senescence (Lee et al., 2015; Zhao et al., 2016). Correspondingly, *pyl duodecuple* mutant is extremely insensitive to ABA-induced leaf senescence (Zhao et al., 2018). Diverse TFs contribute to ABA-mediated leaf senescence. ABIG1 is induced by drought and ABA, and relays drought through ABA signal to promote leaf senescence (Liu et al., 2016). ABA-responsive element (ABRE)-binding TFs, ABF2/ABF3/ABF4 directly activate expression of chlorophyll catabolic enzyme genes (*NYE1, NYC1*, and *PAO*) and SAGs to mediate ABA-triggered leaf senescence and chlorophyll degradation (Gao et al., 2016). Similarly, OsNAP and ONAC054 which are induced by ABA promote the onset and progression of leaf senescence via positively regulating chlorophyll degradation and SAGs (Liang et al., 2014; Sakuraba et al., 2020), thus OsNAP and ONAC054 link ABA to leaf senescence by fine-tuning expression of SAGs. ABI5 acts as another core regulator in ABA-mediated leaf senescence. In apple, MdBBX22, MdWRKY40, and MdbZIP44 all interact with MdABI5 to delay or promote leaf senescence via repressing or enhancing its transcriptional activity (An et al., 2021b). The LEA protein, ABR, is also regulated by ABI5 involving in dark-induced leaf senescence (Su et al., 2016).

As a stress-responsive hormone, ABA mediates the environmental stress-induced leaf senescence process. *AtMYBL* substantially expresses in old leaves, and is also induced by ABA and salt stress. Overexpression of *AtMYBL* displays an enhanced leaf senescence with corresponding changes of chlorophyll content, ion leakage and SAGs expression (Zhang et al., 2011). As an ABA-induced transcription factor, NTL4 mediates drought-induced leaf senescence by promoting ROS production in *Arabidopsis* (Lee et al., 2012). VND-INTERACTING 2 (VNI2), an ABA-responsive NAC transcription factor, integrates ABA-associated abiotic stress signals into modulation of leaf longevity by regulating a subset of *COR* and *RD* genes (Yang et al., 2011). Additionally, ABA regulates leaf senescence by interplaying with other phytohormones. For example, ABA antagonizes CKs-delayed leaf senescence by upregulating the expression of *OsCKX11*, which catalyzes the degradation of CKs in senescing leaves (Zhang et al., 2021c). Collectively, ABA is a key regulator for integrating environmental stress signals into leaf senescence regulation, and is an important target for improving crop yield and quality through molecular breeding.

### Brassinosteroids

Brassinosteroids (BRs) are a class of plant-specific steroid hormones, regulating many aspects of plant physiological processes, such as shoot, root, leaf development, and resistance to biotic stress (Peres et al., 2019). BRs accelerate leaf senescence in a dose-dependent manner (Saglam-Çag, 2007). The application of higher doses of exogenous epibrassinolide promotes leaf senescence, increases peroxidase activity and decreases chlorophyll content in wheat leaves. According to some studies on endogenous BR homeostasis, the stimulating effect of BR on leaf senescence was also elucidated. DET2 encodes a steroid 5α-reductase, and the delayed leaf senescence associated with an apparent phenotype of *det2* mutants in the brassinolide biosynthetic pathway may be due to the elimination of BR biosynthesis (Li et al., 1996). UGT73C6 was identified as an enzyme that catalyzes BR glucosylation and inactivates BR in the phytoplankton. Consistently, overexpression of *UGT73C6* delays leaf senescence (Husar et al., 2011). Transgenic plants overexpressing *P450su1*, which encodes the *CYP105A1* monooxygenase gene disrupt BR signaling by inactivating BRs, display the delayed senescence phenotype (Dasgupta et al., 2011). In addition, a dominant mutant *drl1-D* exhibited prolonged senescence as the endogenous levels of several BRs were significantly reduced (Zhu et al., 2013). The DRL1/BAT1 gene encodes an acyltransferase that catalyzes the conversion of BR intermediates to inactive conjugates via esterification. Transgenic creeping bentgrass overexpressing *AtBAT1* also showed delayed senescence (Han et al., 2017). These results suggest functional manipulation of BR levels to improve agronomic traits by regulating leaf senescence in dicot and monocot crops.

BR is perceived by leucine-rich repeat receptor kinase BRI1 (BRASSINOSTEROID INSENSITIVE 1) to induce signal transduction. The *bri1* exhibits a delay in leaf senescence, supporting a positive role of BR in senescence (Li and Chory, 1997). BES1 (BRI1 EMS SUPPRESSOR 1), a TF of BR signaling, accumulates in the nucleus in response to BR and accelerates leaf senescence once BES1 is activated (Yin et al., 2002). BRI1-associated kinase1 (BAK1), as a part of BR receptor complex, mediates BR-dependent responses. BAK1-LIKE 1 (BKK1) and BAK7, the homologous of BAK1 are respectively reported to act redundantly with BAK1, however *bak1 bkk1* and loss function of both *bak1* and *bak7* display early senescence with upregulated SAGs (He et al., 2007; Jeong et al., 2010). These phenomena suggest that a BR-independent pathway involves in BAK1, BKK1, and BAK7-mediated senescence. Recently, ATBS1-INTERACTING FACTOR 2 (AIF2), a non-DNA-binding bHLH TF has been demonstrated to retard dark-triggered and BR-induced leaf senescence. BR-induced reduction of AIF2 protein associates with the promotion of leaf senescence (Kim et al., 2020). In summary, BR as an important growth-related hormone, its regulation of leaf senescence is always closely linked to the leaf development phenotype, so it may be integrated with other factors, similar to CK and auxin signaling to regulate age-induced or environmental stimulus-induced leaf senescence.

### Strigolactones

Strigolactones (SLs) are a group of terpenoid lactones consisting of a tricyclic lactone and hydroxymethyl butanolide. SLs are well known as communication signals for parasitic and symbiotic interactions, and they were first identified to function as phytohormones to inhibit shoot branching in plants (Yamada and Umehara, 2015; Omoarelojie et al., 2019). The role of SLs in regulating plant growth and development was investigated, and the enhancement of leaf senescence by SLs was gradually elucidated. MAX3/RMS5/D17/DAD3 and MAX4/RMS1/D10/DAD1 encode carotenoid cleavage dioxygenases 7 (CCD7) and 8 (CCD8) respectively, involving in biosynthetic reactions of carlactone, the key precursor of SL. Interestingly, a reduction in expression of *CCD7* and *CCD8* results in delayed leaf senescence (Snowden et al., 2005; Ledger et al., 2010; Liu et al., 2013; Ueda and Kusaba, 2015), correlating with a positive role of SLs in senescence. *ORE9*, identical to *MAX2*, encoding an F-box protein of SL signal pathway, functions in degrading target proteins through ubiquitin-dependent proteolysis, and *ore9/max2* exhibits increased leaf longevity (Woo et al., 2001; Stirnberg et al., 2007). The orthologous of MAX2/ORE9 in rice, D3, disruption of which also delays leaf senescence with lower decrease of chlorophyll content and membrane ion leakage compared to wild type (Yan et al., 2007). A key characteristic of SLs regulation in leaf senescence is the coordination with other hormones and environmental cues. Leaf senescence was not affected by the application of GR24 (an artificial SL analog), however, once ethylene was present, GR24 strongly enhanced senescence (Ueda and Kusaba, 2015). *MAX3* and *MAX4* genes are drastically induced by ethylene, and SLs biosynthesis mutants such as *max1, max3*, and *max4* show a delayed senescence phenotype in the presence of ethylene (Ueda and Kusaba, 2015), indicating that ethylene mediates SLs biosynthesis during senescence. Furthermore, ENHANCED DISEASE RESISTANCE 1 (EDR1) mediates a phenotype of ethylene-induced senescence, which can be suppressed by mutation in ORE9/MAX2 (Tang et al., 2005). Therefore, this suggests that SLs are likely to accelerate leaf senescence following ethylene signaling. In numerous plants, the levels of endogenous SLs are elevated under conditions of phosphate deficiency (Yamada et al., 2014; Yamada and Umehara, 2015). In rice, SLs-deficient mutants were overly sensitive to GR24 application promoting leaf senescence when assessing chlorophyll content compared with adequate phosphate conditions. It was reported by Yamada et al. (2014) that SLs integrate with nutrient signals to regulate leaf senescence. The similar findings were described in the study that the addition of exogenous sugars alleviated SL-induced senescence in bamboo leaves under dark conditions (Tian et al., 2018). Analysis of transcription abundance in *max1* mutant during an extended night also deciphers a valuable association of carbon starvation and SLs signal in regulating leaf senescence (Xu et al., 2021). In conclusion, SLs are an important class of plant hormones that integrate multiple signals in the regulation of leaf senescence. Identification of more direct downstream targets of the SLs pathway, especially MAX2, is of great interest to elucidate the molecular mechanisms of SLs in leaf senescence.

## Peptide Hormones That Regulate Leaf Senescence

Intercellular communication is important to coordinate the growth and developmental programs of multicellular organisms. In plants, classical phytohormones associated with small lipophilic compounds, such as auxin, CKs, GAs, ABA, and ethylene, greatly contribute to intercellular interactions involving different aspects of growth and development. In addition to classical hormones, researches show that multiple families of small polypeptide signaling molecules also play crucial roles in cell-to-cell interaction (Kende and Zeevaart, 1997; Matsubayashi and Sakagami, 2006). These secretory or non-secretory peptides regulate plant growth and development, and responses to environmental stresses, including defense responses, shoot meristem maintenance, root growth, leaf-shape regulation, nodule development, and organ abscission (Matsubayashi and Sakagami, 2006; Marmiroli and Maestri, 2014; Grienenberger and Fletcher, 2015). For examples, systemin induces production of proteinase inhibitors I and II, and plays obvious roles in systemic wound responses in distal leaves (Pearce et al., 1991; Lee and Howe, 2003). RALF (Rapid ALkalinization Factor) can cause alkalinization of the culture medium and a concomitant activation of an intracellular mitogen-activated protein kinase (Pearce et al., 2001). With the progress of researches, RALFs were unraveled to regulate myriad physiological processes, such as root growth and development, root hair size, pollen tube growth, polytubey block, salt stress, and so on (Pearce et al., 2001; Mecchia et al., 2017; Zhu et al., 2020; Zhao et al., 2021; Zhong et al., 2022). These results strengthen the importance of peptides' functions in plants. As the biological activities and functions of these peptide molecules are understood, they are considered to be “peptide hormones”. However, the function of peptide hormones in leaf senescence is largely unknown.

**Figure 1:**
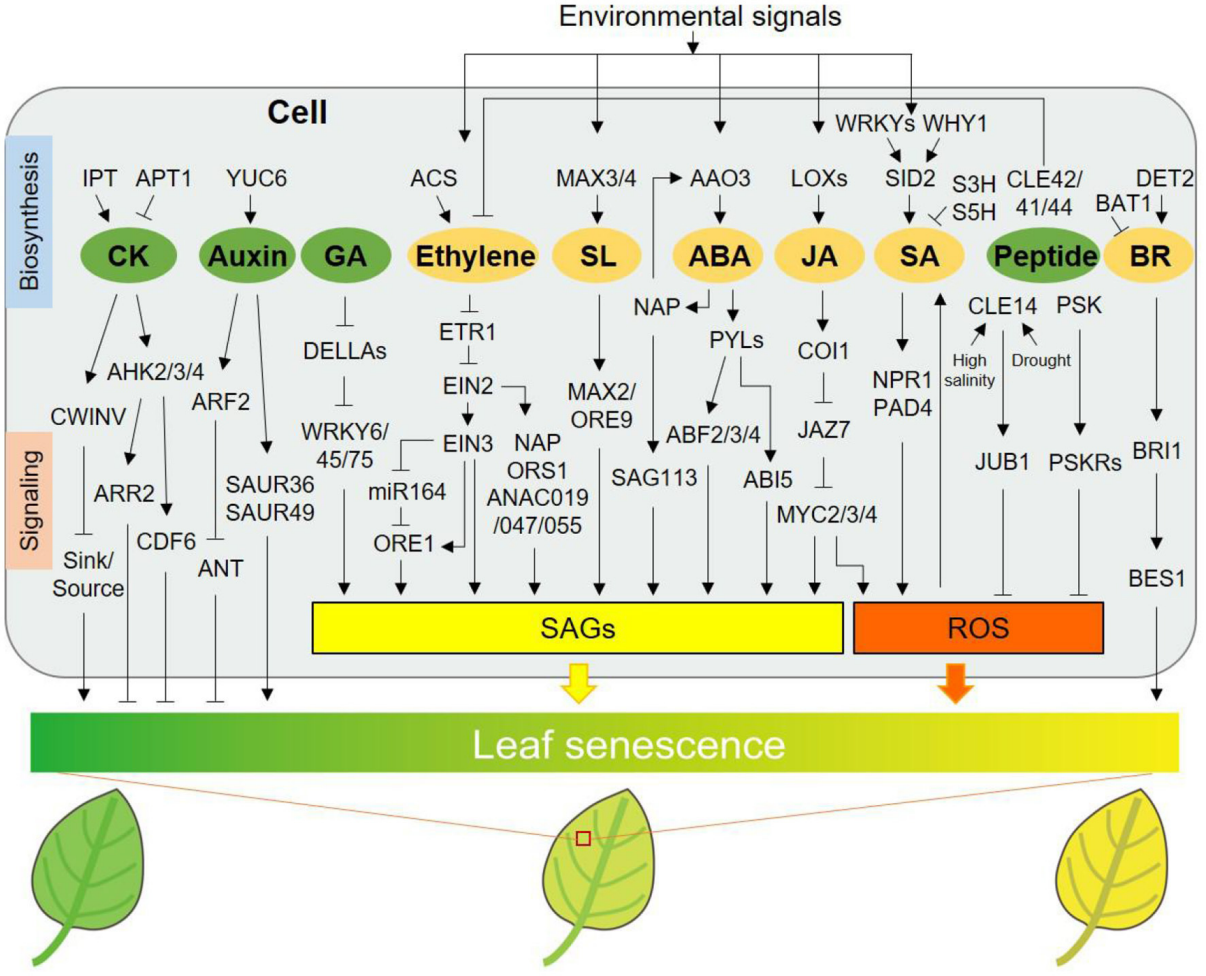
Regulation of leaf senescence by classical and peptide hormones. IPT and APT1 participate in CK biosynthesis and catabolism, respectively. AHK2/3/4, ARR2, and CDF6, signaling components of CK, delay leaf senescence. CWINV (cell-wall invertase) delays senescence via regulating sink-source relations. YUC6 delays leaf senescence by increasing auxin biosynthesis, while ARF2 and SAUR36/49 promote leaf senescence by transmitting auxin signal. ANT, a downstream component of ARF2, postpones senescence phenotype. DELLA proteins delay processes of GA-induced leaf senescence via inhibiting functions of various WRKYs. ACS is involved in ethylene biosynthesis. ETR1, one receptor of ethylene signaling pathway, is involved in ethylene-induced leaf senescence. Ethylene promotes leaf senescence through EIN2-EIN3-miR164-ORE1 pathway or several EIN2 downstream components, including NAP, ORS1, and ANAC019/047/055. SL accelerates leaf senescence *via* functions of MAX2/ORE9. NAP can elevate ABA biosynthesis via inducing AAO3 expression, and ABA-NAP-SAG113 pathway promotes leaf senescence. The receptors of ABA, PYLs and the downstream TFs, ABF2/3/4 and ABI5 all promote ABA-triggered leaf senescence. LOXs promote leaf senescence by increasing JA content under stress conditions. JA promotes senescence via signaling pathway relating to COI1, JAZs, and MYC2/3/4, with increased expression of *SAG*s and enhanced ROS. WHY1 and several WRKYs promote SA content through elevating expression of SID2, a key synthase for SA biosynthesis. S3H and S5H catalyze SA to decline activated form of SA. SA promotes leaf senescence dependent on NPR1 and PAD4, associated with ROS. Peptide hormones including CLE42/41/44, CLE14, and PSK delay leaf senescence. CLE42/41/44 function redundantly to delay senescence via antagonizing with ethylene pathway. CLE14 is induced by high salinity and drought stresses and reduces ROS level via transcriptional activation of JUB1, a NAC TF. PSK may be perceived by its receptor PSKRs to contribute to ROS scavenging. In addition, GA, ethylene, SL, and ABA are also associated with regulation of a series of *SAG*s expression. DET2 contributes to BR biosynthesis and BAT1 inactivates BR. BR accelerates leaf senescence through signaling transduction involving positive regulators, BRI1 and BES1. Hormones including ethylene, SL, ABA, JA, and SA play significant roles in integrating environmental signals into the regulation of leaf senescence. Hormones presented in green ellipses including CK, auxin, GA, and mentioned peptides delay leaf senescence, whereas ethylene, SL, ABA, JA, SA, and BR in orange ellipses promote leaf senescence, according to phenotypic changes caused by exogenous application. SAGs, senescence-associated genes; ROS, reactive oxygen species.

Recently, several researches provide insight into the regulatory mechanism of peptide hormones in leaf senescence. The small secreted peptide CLE14 (CLAVATA3/ESR-RELATED 14) postpones leaf senescence by transcriptional activation of JUB1-dependent ROS scavenging genes (Figure 1), leading to reduced ROS level in leaves (Zhang et al., 2022b). CLE14 is significantly induced by age, high salinity, drought, ABA, SA, and JA, thus it acts as a “brake signal” to modulate age-dependent and stress-induced leaf senescence (Zhang et al., 2022b). In another work, CLE42 delays leaf senescence by antagonizing ethylene signaling pathway. CLE42 suppresses ethylene biosynthesis and increases the accumulation of EBF proteins, sequentially resulting in the decreased function of EIN3. Additionally, CLE41/44 function redundantly with CLE42 to regulate leaf senescence (Zhang et al., 2022a). The peptides are usually recognized by membrane-localized receptors and transduce the signaling responses. It was reported that the CLE41/44, also called TDIF, could be bound by receptor TDR/PXY, a leucine rich repeat receptor-like kinase (LRR-RK) (Fisher and Turner, 2007; Hirakawa et al., 2008). With the help of co-receptor SERK, the TDIF-TDR/PXY signaling plays an important role in plant vascular development (Zhang et al., 2016). Whether these receptors also take part in leaf senescence regulation is an interesting question for investigation. Especially, beyond the role of CLE41/CLE44 themselves in senescence, the TDIF-TDR/PXY can lead to inactivation of BES1 TF, a crucial regulator of senescence-promotion hormone BR, through regulation of GSKs activity (Kondo et al., 2014). The known CLE receptors are limited, it is necessary to find more CLE receptors for better understanding their functions in leaf senescence. In addition to CLE peptides, PSK (Phytosulfokine) and PSY1 are also involved in the regulation of leaf senescence. The membrane-localized PSKR is the receptor of PSK, and loss-of-function *pskr1-1* mutant exhibits premature leaf senescence, which is consistent with a delayed effect of exogenous PSK-α on senescence (Yamakawa et al., 1999; Matsubayashi et al., 2006). Interestingly, two other homologs of PSKR also encode LRR-RKs, one of which acts on PSY1 perception and has an overlapping function with PSKR; thus, the triple mutant shows an enhanced senescence phenotype compared to the single *pskr1* mutant (Amano et al., 2007). Posttranslational modification of small peptides is essential for their biological functions (Matsubayashi, 2014). Biological activities of PSK and PSY1 need tyrosine sulfation, catalyzed by transmembrane tyrosylprotein sulfotransferase (TPST). Interestingly, loss-of-function of TPST accelerates leaf senescence (Komori et al., 2009). PSK may influence leaf senescence through protective action in chlorophyll content under heat stress (Yamakawa et al., 1999), or delays senescence by elevating expression of ROS scavenging genes and reducing endogenous H_2_O_2_ accumulation during storage of fruits and flowers (Aghdam et al., 2021a,b). In summary, peptide hormones interact with classical hormones to regulate leaf senescence. Hundreds of peptide hormones and putative peptide molecules have been identified in plants, and their functions in the regulation of leaf senescence deserve further exploration.

## Conclusions and Perspectives

**Figure 2:**
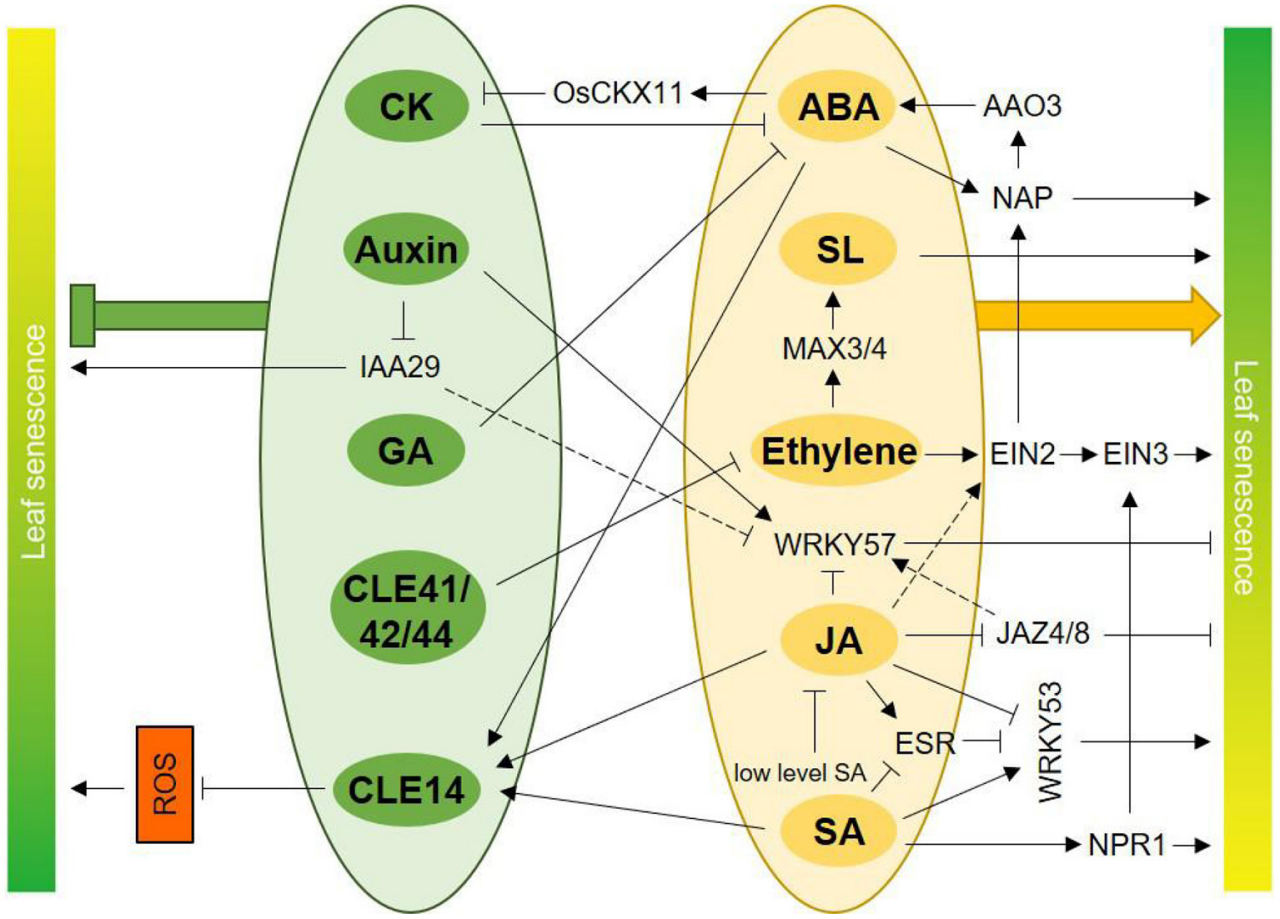
Interplays among classic and peptide hormones in senescence regulation. CK inhibits ABA biogenesis and ABA represses CK content through elevating expression of *OsCKX11*. GA treatment inhibits the increase of ABA content during leaf senescence. *CLE14* is induced by ABA, JA, and SA, and CLE14 act as a “brake signal” to these hormones-induced leaf senescence though repressing ROS level. CLE41/42/44 delay leaf senescence by antagonizing with ethylene signaling pathway. Ethylene promotes SL biosynthesis via inducing expression of *MAX3* and *MAX4* during senescence, so ethylene and SL coordinately regulate leaf senescence. Phytohormone ABA and the core component of ethylene, EIN2, induce the expression of NAP, a positive regulator of leaf senescence. Conversely, NAP increases ABA biosynthesis through inducing expression of *AAO3*. JA-induced leaf senescence is dependent on EIN2. WRKY57 is a negative regulator of JA-induced leaf senescence. JA inhibits accumulation of WRKY57 protein and auxin promotes WRKY57, so that WRKY57 acts as an integrator of JA and auxin. Besides, the repressors of JA and auxin signaling pathway, JAZ4/8 and IAA29 both interact with WRKY57, which may be another regulatory level of interplay between JA and auxin. Ethylene and SA synergistically accelerate leaf senescence through interaction of NPR1 and EIN3 and a concomitant promotion of *SAGs* expression. SA represses the JA-inducible protein ESR, and ESR inhibits the functions of WRKY53, a positive regulator of leaf senescence. Furthermore, JA reduces the expression of *WRKY53* and SA induces *WRKY53* oppositely. Thus, ESR and WRKY53 are integrators of antagonism between JA and SA in leaf senescence. Moreover, low concentration of SA can alleviate JA-induced leaf senescence. Generally, hormones presented in green ellipses including CK, auxin, GA, CLE41/42/44, and CLE14 delay leaf senescence (bold green symbol). Hormones in orange ellipses including ABA, SL, ethylene, JA, and SA promote leaf senescence (bold orange arrow).

Leaf senescence is a highly coordinated process controlled by a complex network of genes. The classical plant hormones and peptide hormones contribute significantly to the regulation of the initiation and progression of leaf senescence (Figure 1), which underlies the orderly degradation and macromolecular degradation of chloroplasts and is closely linked to the maximization of nutrient utilization for growth and development. Hormones can precisely regulate leaf senescence, thanks to the flexibility of their action. CKs, auxins and GA are known to delay leaf senescence, while ethylene, SA, JA, ABA, BRs and SLs promote senescence, and even peptides can regulate senescence in a viable manner. However, when referring to the role of a certain hormone in the regulation of leaf senescence, we must be aware of the dosage effect. Hormones at low concentrations delay leaf senescence, but high concentrations promote senescence (Song et al., 2016). This is partly attributed to the interactions between hormones (Saglam-Çag, 2007). Hormone-mediated leaf senescence involves signal transduction pathways and a myriad of transcriptional regulations, yet an increasing number of studies have reported multi-level gene regulation of leaf senescence. For example, post-transcriptional alternative splicing regulation of ONAC054 involves in ABA-induced leaf senescence (Sakuraba et al., 2020). Although ONAC054α is only induced by ABA, its alternative splicing form ONAC054β is induced by ABA and high concentration of ACC, thus the multilayered regulation provides more available regulatory nodes for hormonal interplays. The interaction between plant hormones is another advantage of their precise regulation of leaf senescence under different environmental conditions, which is crucial for the operation of agronomic improvement. The interplays among classic and peptide hormones in the regulation of leaf senescence was summarized in Figure 2. Understanding the functions of key genes or proteins that link different hormone signals will help us to understand the intrinsic regulation of leaf senescence by hormone signaling networks. In addition, hormones play an important role in integrating environmental signals into specific components or pathways associated with leaf senescence. In conclusion, dissecting the novel functions of hormone signaling components in leaf senescence is worth further attention.

The integration of plant hormones affecting the process of leaf senescence and environmental factors is more easily achieved in traditional experimental systems. Nevertheless, the initiation of leaf senescence depends on age-related factors, which is a major part of the mystery explored. Although hormones, for instance ethylene, cannot affect leaf senescence until leaf age reaches a certain developmental stage (Jing et al., 2002), they ensure the regulation of plant growth and development, which may contribute to the accumulation of age factors that alter the onset of aging. The initiation of senescence is not uniform, as leaf cells are usually in different states of senescence over a certain period of time. Taking advantage of single-cell sequencing, combined with spatiotemporal transcriptome analysis, offers the possibility to address the challenges of senescence research, leading to a better understanding of the initiation of senescence and the corresponding hormonal functions during this process.

Peptide hormones consist of a family of different classes of peptides that regulate plant growth, development and response to environmental stresses. So far, CLEs and PSK have been described as substances that regulate leaf senescence (Matsubayashi et al., 2006; Zhang et al., 2022a), and more peptide signaling molecules deserve to be explored to expand the understanding of leaf senescence. LRR-RKs work redundantly in perception of PSK and PSY1, and sometimes one LRR-RK does not only recognize one particular peptide (Amano et al., 2007), suggesting difficulties in finding novel peptide components and establishing explicit signaling transduction. Distinct from forward genetic screening and reverse genetic analysis, chemical genetic screening can overcome the problem of gene functional redundancy and is a good option to further explore more components of leaf senescence. With the establishment of more complex hormonal signaling pathways associated with leaf senescence, we expect that the regulatory mechanisms of leaf senescence will provide important clues for improving crop yield and quality.

## Author Contributions

ZL, HG, and PH conceived and designed this review. PH wrote the manuscript. All authors have read and agreed to the published version of the manuscript.

## Funding

This work was funded by grants from the Shenzhen Science and Technology Program (No. KQTD20190929173906742 to HG), the National Natural Science Foundation of China (No. 32170345 and 31970196 to ZL), the National Key Research and Development Program of China (No. 2019YFA0903904 to HG), and the startup funding for plant aging research from Beijing Forestry University (ZL).

## Conflict of Interest

The authors declare that the research was conducted in the absence of any commercial or financial relationships that could be construed as a potential conflict of interest.

## Publisher's Note

All claims expressed in this article are solely those of the authors and do not necessarily represent those of their affiliated organizations, or those of the publisher, the editors and the reviewers. Any product that may be evaluated in this article, or claim that may be made by its manufacturer, is not guaranteed or endorsed by the publisher.
